# Detection of Hypoglycin A and MCPrG Metabolites in the Milk and Urine of Pasture Dairy Cows after Intake of Sycamore Seedlings

**DOI:** 10.1021/acs.jafc.3c01248

**Published:** 2023-07-07

**Authors:** Anna Maria Engel, Ahmed H. El-Khatib, Fenja Klevenhusen, Michael Weiss, Sabine Aboling, Benjamin Sachse, Bernd Schäfer, Stefan Weigel, Robert Pieper, Carola Fischer-Tenhagen

**Affiliations:** † Department Safety in the Food Chain, German Federal Institute for Risk Assessment (BfR), 10589 Berlin, Germany; ‡ Faculty of Organic Agricultural Sciences, University of Kassel, 37213 Witzenhausen, Germany; § Institute for Animal Nutrition, University of Veterinary Medicine Hannover, Foundation, 30173 Hannover, Germany; ∥ Department Food Safety, German Federal Institute for Risk Assessment, 10589 Berlin, Germany; ⊥ Center for Protection of Laboratory Animals, German Federal Institute for Risk Assessment (BfR), 12277 Berlin, Germany

**Keywords:** secondary plant metabolites, food safety, ruminants, observational, milk, LC/MS−MS, plant toxin, poisoning

## Abstract

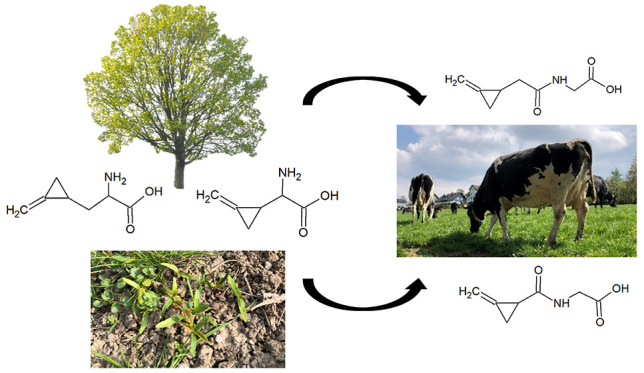

Hypoglycin A (HGA), methylenecyclopropylglycine (MCPrG), hypoglycin B (HGB), and γ-glutamyl-α-(methylenecyclopropyl) glycine (γ-glutamyl-MCPrG) are secondary plant metabolites occurring in sycamore maple (*Acer pseudoplatanus*) as well as several other Sapindaceae (e.g., *Blighia sapida*). By interfering with energy metabolism, they may cause severe intoxication in humans and other species. However, to date, there is not enough data available concerning the intake, metabolism, or excretion of sycamore maple toxins in dairy cows. In May 2022, five cows were observed over four days, when they had first access to a pasture with two sycamore maples. Grazing of their seedlings that grew numerously in between the pasture plants was monitored by direct observation. Milk samples were drawn both from individual cows and from the bulk tank. Spontaneous urine samples were collected from all cows on day 3 after access to the pasture. Seedlings (100 g) were sampled on the pasture and analyzed, together with milk and urine samples, for sycamore toxins and their metabolites using liquid chromatography–tandem mass spectrometry and liquid chromatography-high-resolution mass spectrometry. Cows ingested sycamore seedlings while grazing. Values of HGA in milk were below the limit of quantification. However, metabolites of HGA and MCPrG were detected in individual milk samples already at the end of the first day of grazing. Urine samples of all five cows showed higher concentrations of conjugated HGA and MCPrG metabolites than in milk. Observations suggest that dairy cows may have a low susceptibility toward sycamore maple toxins. However, whether this could be attributed to foregut fermenting species in general requires further elucidation.

## Introduction

Exposure to non-proteinogenic amino acids hypoglycin A (HGA) and methylenecyclopropylglycine (MCPrG) can lead to severe intoxication in several species, including humans,^1,2^ horses,^3,4^ deer,^5,6^ gnus,^7^ and camels.^8^ As secondary plant constituents of the soap tree family (Sapindaceae), these substances have been found in fruits of litchi (*Litchi chinensis*),^9^ akee (*Blighia sapida*),^1,10,11^ and various maple trees including sycamore maple (*Acer pseudoplatanus*).^3,12−14^ Ingestion of seedlings and seeds of sycamore maple trees is known to cause poisoning in grazing horses, resulting in the so-called atypical pasture myopathy (AM) characterized by muscle stiffness, myoglobinuria, frequently found hyperglycemia, and mortalities.^4,15,16^ Contrary to horses, studies conducted in humans or laboratory animals reveal hypoglycemia following the ingestion of maple toxins.^2,17−19^

Additionally, hypoglycin B (HGB) and γ-glutamyl-MCPrG have been detected in seeds^13^ and seedlings^20^ of sycamore maple trees. However, data in rabbits, monkeys, or rats indicate a lower hypoglycemic activity of HGB compared to HGA.^21^ Noteworthily, HGB and γ-glutamyl-MCPrG are barely addressed in scientific publications dealing with sycamore intoxications in farm animals, albeit their co-occurrence is relatively likely.

Regarding the mode of action of HGA and MCPrG, it is known that metabolites of both compounds disrupt fatty acid metabolism and, thus, cause an interference with energy metabolism.^22−25^ Following the formation of the intermediates methylenecyclopropylpyruvate and methylenecyclopropylglyoxalate from HGA and MCPrG, respectively, and conversion to methylenecyclopropylacetate (MCPA) or methylenecyclopropylformate (MCPF), conjugation with coenzyme A (CoA) occurs, leading to the formation of the metabolically active forms methylenecyclopropylacetyl-CoA (MCPA-CoA) and methylenecyclopropylformyl-CoA (MCPF-CoA) for HGA and MCPrG, respectively.^26^ These compounds are potent inhibitors of acyl-CoA dehydrogenases and enoyl-CoA hydratases enzymes involved in β-oxidation—resulting in excretion of incomplete degradation products of fatty acids in urine and an altered acylcarnitine profile in blood.^26−28^ The metabolites MCPA and MCPF are conjugated with carnitine or glycine and may be excreted via the renal system. The presence of these metabolites in urine and blood of affected horses and humans is considered a biomarker of exposure to HGA and MCPrG.^4,9^

Recently, the detection of HGA traces in duplicate samples from one bulk milk tank of a dairy farm raised concerns that ingestion of sycamore seeds by dairy cows may pose a risk for animals and consumers.^29^ However, to our knowledge, there is neither a direct proof that dairy cows ingest sycamore seedlings nor are there data on metabolism and excretion of maple toxins by dairy cows available. For ruminants in general, toxic effects caused by HGA/MCPrG have only been demonstrated in the browsing Père David’s deer, gnus, and Bactrian camels,^5−8^ while there are no known cases of poisoning in grazers like sheep or intermediate types like goats.^30,31^ Gonzalez-Medina et al.^32^ detected HGA in serum samples from ewes and their lambs and also MCPA conjugates in one ewe at 0, 2, and 7 days after grazing on a pasture with sycamore seedlings. No animal in this case showed any adverse effects, suggesting a low (or even no) sensitivity for the toxins in sheep. This observation supports the assumptions of differences in toxicological susceptibility between various ruminant species. Additionally, the detection of HGA traces in the serum of lambs of the abovementioned ewes suggests that the compound may also be transferred into the milk of ewes as it has already been reported for mare’s milk.^33^ Physiological and gastrointestinal peculiarities in ruminants could result in low sensitivity to the toxic effects of certain plant toxins.^34^ For example, due to the large size of the rumen as well as the long retention time, ruminal transformation of HGA might occur before entering the proximal small intestine as the site for HGA absorption.^7,35^ Firstinsights into the effects of the rumen microbiome on detoxification, although, showed no significant decrease in HGA concentrations but rather an increase of yet unknown cause over a short period of 2 h of in vitro incubation in ruminal fluid of adult sheep.^32^

Therefore, the purpose of this study was to investigate whether (1) dairy cows voluntarily ingest sycamore seedlings, and if so, (2) ingestion would result in excretion of HGA, MCPrG, HGB, γ-glutamyl-MCPrG or metabolites in milk or urine, or (3) cows develop certain clinical signs such as those described in AM horses.

## Materials and Methods

### Ethics Statement

All procedures were in accordance with national and international guidelines for animal welfare. Owners gave informed consent for their cows’ inclusion in the study and were asked if they agreed to systematic observation of the animals on their habitual pasture. Cows were physically evaluated by a veterinarian daily. Collection of milk and urine samples followed routine milk performance checks and on the basis of routine veterinary diagnostics by a veterinarian. Owners agreed to maple toxin analysis in samples. The study was permitted by the institute’s animal welfare officer. Therefore, approval was not required for this observational study as treatments were not applied to animals as confirmed by the animal welfare officer of the German Federal Institute for Risk Assessment.

### Animals and Video Recording of Cows

In May 2022, five (Holstein-Frisian × Jersey × Norwegian Red) cows (two primiparous and three multiparous, Cow 1–Cow 5(C1–C5)), approximately 207 ± 7 days in milk, with an average milk yield of 21.3 ± 6 kg, out of a herd of 87 cows, were kept on a pasture (1800 m^2^) with two sycamore maple trees and numerous seedlings growing among the grass. This was the first time in 2022 that the cows were allowed to graze on that pasture. Therefore, an earlier contact with sycamore seedlings from that pasture was unlikely. The pasture was exclusively available for the five cows from 11 am to 3 pm over four consecutive days, while the remaining 82 cows were grazed on a neighboring pasture with visual contact. Cows had ad libitum access to water, which is delivered by a groundwater pump, and received a partial mixed ration (71% grass silage, 10.1% maize silage, 10.1% beet pulp, 2.6% wheat, 2.6% grain maize, 1.6% straw, 1.0% protein press cake, 0.3% minerals, 0.3% bicarbonate, and 0.2% fermented cereals) ad libitum on the feed ally in the barn apart from grazing times. Additionally, a concentrate mixture was provided in the barn in a separate feeding trough, transponder-controlled for each cow, to meet the energy requirements for individual milk yields. During the time on pasture, animals were continuously observed by two independent observers. If spotted, uptake of seedlings was documented by video recording (2 × SONY Handycam HDR-CX240, Apple iPhone 8).

### Collection of Seedling and Vegetation Samples

**Figure 1:**
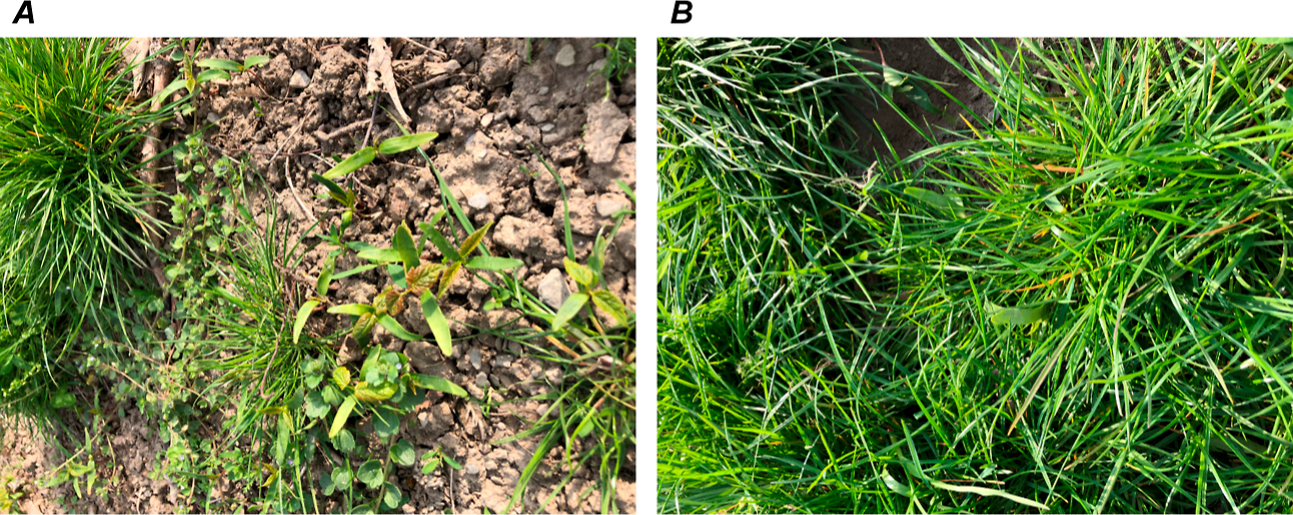
Sycamore maple seedlings with cotyledons and the first pair of leaves on the pasture. (A) Open area and (B) among grasses.

Samples of *A. pseudoplatanus* seedlings were collected on the pasture located in North Rhine-Westphalia, Germany, on the last day (day 4) of the trial. Seedlings of both two-leaf as well as four-leaf stages were sampled representatively in the open area directly under the trees as well as among the grasses (Figure 1).

Altogether, 100 g of seedlings was air-dried at room temperature, homogenized (approx. 500 μm) using knife/ball mills (Retsch, Haan, Germany), and stored under dry conditions at room temperature for subsequent sycamore maple toxin analysis. Additionally, 500 g of seedlings and remaining vegetation were collected and stored at −20 °C until nutrient and chemical analysis.

### Milk Sampling

Cows were milked twice daily at 6:30 am and 6:30 pm. Milk samples were obtained uniformly to represent milk composition from milk letdown to emptying following routine milk performance checks with milk meters (TRU-Test datamars, Auckland, New Zealand) in a fishbone milking parlor (Westfalia/Gea Germany, Düsseldorf, Germany). Samples were available from all 5 animals from the first morning before their release on the pasture (day 1), the first evening milking after their first release on the pasture (day 1), as well as on day 2 and 3 from morning and evening milking, along with morning milk of day 4. Therefore, milk from day 1 of the study, that was milked in the morning, served as T0, as cows had no access to the pasture beforehand. Additionally, the tank milk of the entire herd (*n* = 87) was sampled in the morning and evening. Samples were stored at −20 °C and analyzed for HGA, MCPrG, and their respective metabolites.

### Botanical Survey on Experimental Plots

Before the onset of grazing, seven 50 × 50 cm plots were established that represented density and species composition of the pasture vegetation. Four plots contained sycamore seedlings, while the remaining three plots served as the control with no seedlings. Plots with seedlings represented areas with numerous seedlings (*n* = 33/m^2^) as well as areas with few seedlings (*n* = 4/m^2^). Dominating plants were identified as *Taraxacum officinale*, *Trifolium repens,* as well as various Pocaceae. Daily at 10:30 am, plots were photographed (Nikon D90, Apple iPhone 8). Seedlings were counted before cows were allowed to graze.

The proportions of the grazed area were determined for each plot. “Grazed” in the context of this study is defined as visible signs of missing, i.e., ingested plant parts. Other traces of activities on the grass as footprints or damage by cows resting on the ground were ignored. For estimation of the grazed parts of the plots (50 × 50 cm), they were divided into 10 × 10 cm sub-plots. In each sub-plot, remaining vegetation, expressed as the percentage (%) of original 100% coverage, as well as remaining seedlings were checked from day 1 to day 4 in order to examine whether seedlings were eaten by the cows unintentionally along with grasses. To obtain the extent of ingestion of feed plants on the total area, i.e., the utilization of vegetation by the animals, the remaining amount of untouched plants on day 2 to day 4 was compared to the original amount on day 1 (100%).

### Proximate Analyses of Seedlings and Grassland Growth

Biomass of both seedlings and remaining vegetation were analyzed for dry matter (DM), crude ash, crude protein, crude fat, and crude fiber according to VDLUFA (Association of German Agricultural Analytic and Research Institutes) standard methods.^36^

### Toxin Analysis in Seedlings

(*S*)-Hypoglycin A (HGA, purity 85%), HGB (γ-glutamyl-hypoglycin, 98%), MCPrG (97%), and (MCPA)-C (97%) standards were purchased from Toronto Research Chemicals (Toronto, Canada). MCPA-G (97%) and MCPF-G (97%) standards were purchased from IsoSciences (Ambler, PA, USA).

Seedlings were analyzed according to El-Khatib et al.^20^ Additionally, qualitative detection of HGB was achieved by comparison of retention times and spectra in samples with the HGB reference substance. The reference substance was not available at the time of method development and validation. Thus, quantification of HGB in seedlings was not possible at the time of analysis, and only a qualitative detection was carried out once the substance was available. All solvents used in this study were at least of analytical grade. Solvents used for liquid chromatography–tandem mass spectrometry (LC–MS/MS) and high-resolution tandem mass spectrometry (HR-MS/MS) analysis were of LC–MS grade.

Briefly, 5 mL of deionized water was added to 0.5 g of the homogenized plant material (plants with roots), and the mixture was pre-vortexed and placed in the ultrasonic bath for 10 min at room temperature (Sonorex Super, Bendelin, Berlin, Germany). The samples were centrifuged for 10 min at 4000 rpm (Heraeus Megafuge 16, Thermo Fisher Scientific, Waltham, USA). Afterward, the supernatant was filtered (Ahlstrom Folded filters, NeoLab Heidelberg, Germany) and transferred to a new 15 mL tube. The residue was extracted again with 5 mL of deionized water, centrifuged, filtered, and combined with the first extract. The samples were then measured (undiluted or diluted 1 in 25 with 5% methanol/water) by LC–MS/MS (Q-Trap 6500+, AB Sciex Germany GmbH, Darmstadt, Germany) or LC–HR-MS (QExactive Focus, Thermo Fisher, Dreieich, Germany).

### Toxin Analysis in Milk

Milk samples were analyzed according to El-Khatib et al.^37^ Briefly, 10 mL of milk samples was mixed with 10 mL of 1% formic acid in methanol (v/v). Additionally, 100 μL of formic acid and 1 mL of EDTA solution were added. After shaking the samples in an overhead shaker for 20 min, they were refrigerated at −20 °C for 2 h. Afterward, samples were centrifuged at 4000 rpm and 4 °C for 10 min. The supernatant of the sample was then transferred to a tube containing 0.1 g of the C18 material (Polygoprep 300-30C_18_, Macherey-Nagel, Düren, Germany) and 2 mL of acetonitrile (ACN) and shaken in an overhead shaker for 15 min. Subsequently, samples were centrifuged at 4000 rpm and 20 °C for 10 min. In the case of MCPF-carnitine, the unequivocal confirmation and quantification was not possible due to the lack of a reference standard. However, there is sufficient evidence from mass spectrometric data that MCPF-carnitine was present in samples. Within a mass tolerance of 3 ppm, the accurate mass of MCPF-carnitine was detected by HR-MS. In addition, 3 probable mass transitions for MCPF-carnitine have been monitored and showed the same retention time. The retention time of the tentative MCPF-carnitine signal (3.37 min) lies within the predicted range considering the elution profiles of MCPF-glycine (3.47), MCPA-glycine (3.83), and MCPA-carnitine (3.66). Thus, a qualitative approach was used. To this end, the chromatographic peak areas of tentatively identified MCPF-carnitine were compared to investigate if any trends in levels can be seen in the samples.

### Urine Sampling and Toxin Analysis in Urine

Urine samples were obtained from all 5 cows on the basis of routine veterinary diagnostics upon spontaneous micturition on day 3 at 4.30 pm and afterward analyzed for their levels of HGA, MCPrG, and their respective metabolites by applying the method of El-Khatib et al.^37^ Briefly, the urinary creatinine concentration was measured at an accredited medical analytics laboratory (Labor 28 GmbH, Berlin, Germany). The urine samples were subsequently diluted with 5% MeOH to a creatinine concentration of 0.1 mg/dL and then analyzed by LC–MS/MS.

Urine samples of dairy cows kept and taken care of at the research farm of the German Federal Insitute for Risk Assessment (BfR) served as controls.

### Statistical Analysis

Statistical analysis was carried out using R Version 4.2.1 (2022-06-23)^38^ package MCMCglmm.^39^ A linear mixed effect model was performed using MCMCglmm (MCPA ∼ day + time, random = ∼cow) as the code, where time represents the time point (morning or evening) at which samples were taken. Cows where included as random effects in order to take into account that the animals were repeatedly sampled. Significance was assumed, if *p*-values were below 0.05.

## Results and Discussion

Here, we report, for the first time, the ingestion of sycamore seedlings with measurable concentrations of HGA and MCPrG by dairy cows during grazing on pasture. We detected metabolites of these substances in urine as well as in milk samples. The cows did not show clinical signs such as visible manifestations of illness or discomfort. Nevertheless, possible subclinical changes in acylcarnitine profiles in urine or blood cannot be conclusively excluded.

### Seedling Intake and Nutritional Values

**Figure 2:**
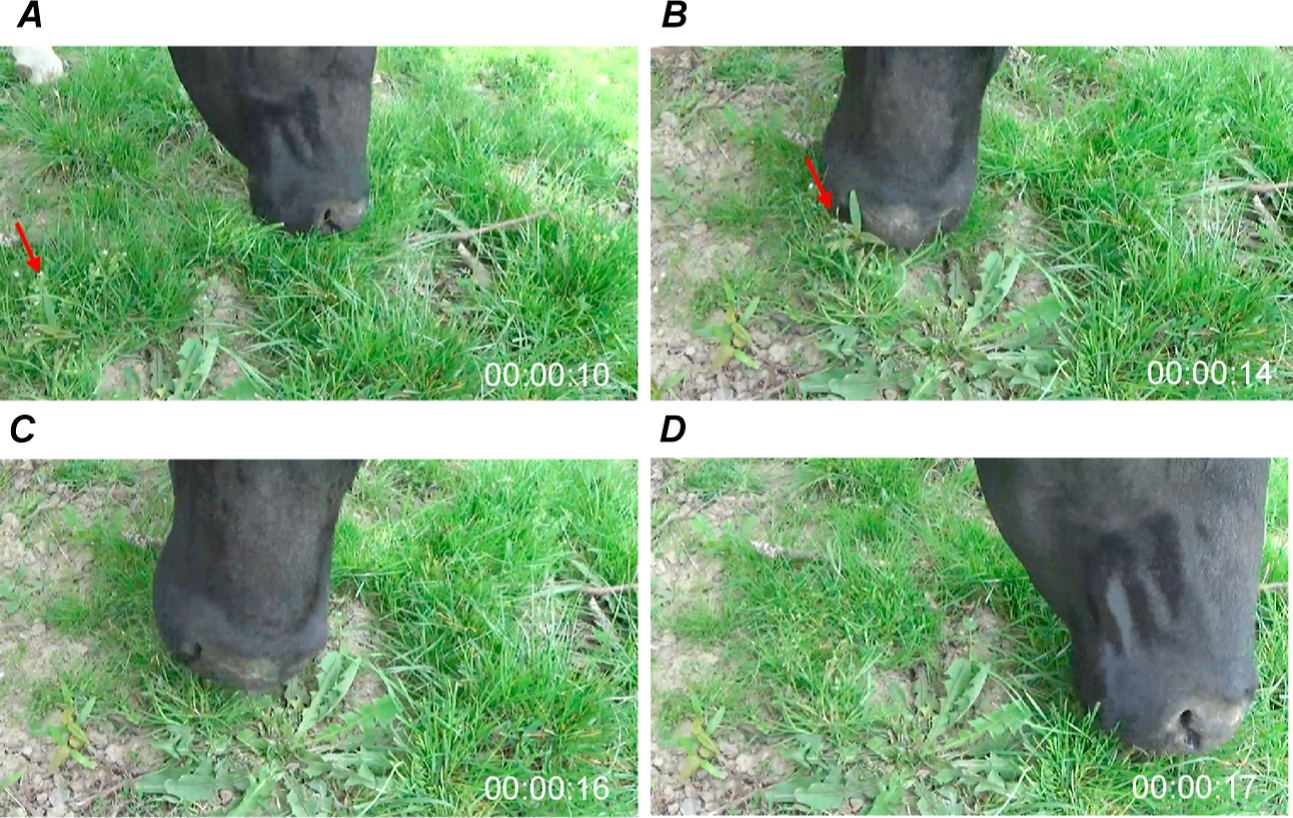
Ingestion of *A. pseudoplatanus* seedling by study cow. (A) Seedling appears in the picture while the cow is grazing. (B) Cow touches the seedling with the mouth. (C) Mouth of the cow is located above the seedling. The seedling is ingested. (D) Seedling is no longer visible.

Already, during the first day of the experiment, an ingestion of sycamore seedlings by the cows was observed visually and also captured on video (Figure 2).

**Figure 3:**
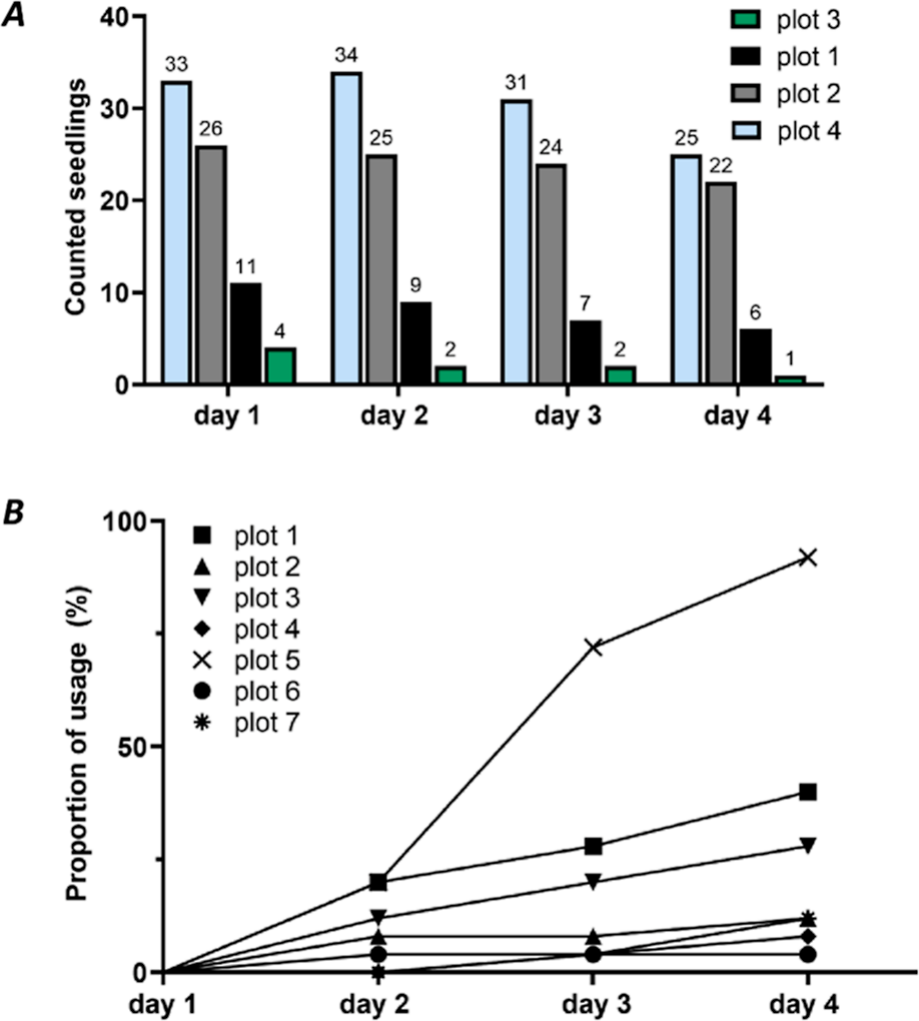
Overview of experimental plots. (A) Counted seedlings (*A. pseudoplatanus*) per plot (50 × 50 cm) per day. Plots were checked and photographed daily for remaining seedlings before moving animals to pasture. (B) Proportion of usage of experimental plot 1 to 7.

In addition, over the 4 day period, the number of seedlings decreased in all experimental plots containing seedlings (plot 1–plot 4), while the relative amount of ingested plant parts of the remaining vegetation increased (plot 1–plot 7) (Figure 3).

There was no difference in the number of missing seedlings of two- and four-leaf stages, indicating that cows, contrary to horses, may not discriminate between seedlings of two- and four-leaf stages.^40^ Since the seedlings were not selected but always eaten along grasses and forbs, we observed their ingestion as a byproduct of grazing. Ghislain et al.^41^ reported that 78–86% of seedlings disappear naturally on a pasture within three to four weeks. Nevertheless, as there is not only a direct proof of consumption on the field level by various distinct field methods but also on the chemical level as metabolites of HGA and MCPrG are further detected in urine and milk samples, the decrease in seedlings in experimental plots likely goes back to ingestion by the cows.

Seedlings contained on average 2.6 ± 0.05 g of HGA/kg of DM and 0.2 ± 0.002 g of MCPrG/kg of DM (Table 1).

**Table 1:** Composition of Sycamore Maple Seedlings and Pasture Grass

		seedlings (g/kg)	pasture grass (g/kg)
dry matter	285	273	
	HGA^a^	2.6	
	MCPrG^b^	0.2	
	HGB^c^^,^^d^	present	
	crude protein	11.6	12.3
	crude ash	29.9^e^	7.6
	crude fiber	6.2	10
	ether extract	1.6	2.5

a Hypoglycin A (HGA).

b Methylenecyclopropylglycine (MCPrG).

c Hypoglycin B (HGB).

d Qualitative detection.

e Data can only be assessed to a limited extent, as contamination with soil is possible due to the root content.

Comparable contents of HGA in sycamore seedlings, 2.1–3.4 and 0.3–2.7 g/kg, have been reported by Baise et al. and Gonzalez-Medina et al., respectively.^14,42^

Hypoglycin B was detected in all plant samples. Crude fiber contents in seedlings (21.6 g/kg of FW) were lower than in the rest of the herbaceous vegetation (36.7 g/kg of FW) (Table 1). Nevertheless, crude fiber contents in seedlings were in agreement with contents for seedlings previously reported by Aboling et al.^40^ Fiber content of the herbaceous vegetation, in Table 1, was expected as sampling was done on the onset of the grazing season with low crude fiber content and increased crude protein content before shooting.^43^ The seedlings in this study showed lower fat and protein content compared to the pasture grass as already described in the literature.^28^

Several reports have shown that herbivores may select their diets either due to nutritional needs or as a strategy to reduce toxin intake.^44,45^

Here, there were no major differences at least between the nutritional value of the seedlings and that of the remaining grass. Since cows were able to meet their energy and nutrient needs through the partial mixed ration and the addition of concentrates offered in the barn, increased intake of either seedlings or grass caused by reduced availability of nutritious feed is not likely in the present study.

Overall, the selective grazing behavior of cows, due to genetic selection for high yielding patterns, is no longer as distinct as in non-domesticated herbivores and is predominantly seen in heterogeneous areas,^46,47^ leading to the assumption that cows in this study could simply not discriminate between seedlings and grass. Freeland and Janzen^48^ reported that cows may include a variety of plant species in their diet up to high everness, so that secondary plant components derived from a single plant variety enter the body below harmful levels.

Referring to studies reporting that Père David’s deer from two Zoo’s in Germany picked up either seedlings and seeds^5^ or actively ingested leaves and seeds of sycamore maple trees,^6^ there is no information on the nutrient supply of the diseased Père David’s deer. Even if species-specific requirements are met, active submission of the plant may result in increased uptake due to inevitably overgrazed areas, boredom of animals, or attractiveness of presented plant parts. In addition, there is no information on whether the animals could have avoided harmful levels of secondary compounds due to complementary feed intake, as described by Freeland and Janzen.^48^ This is in contrast to the study conducted here as well as to the study conducted in ewes and their lambs by Gonzalez-Medina et al.^32^ Nevertheless, since clinical cases rarely occur in enclosures, it must be assumed that the susceptibility of the individual animal species still varies.

Our results prove the hypothesis that cows ingest maple seedlings with high levels of maple toxins when seedlings are present on the pasture. It is however not clear whether animals could simply not discriminate seedlings and pasture plants by taste or smell or if there is indeed an avoidance strategy up to a certain tolerance level by taking up a variety of grasses and plants, as postulated by Freeland and Janzen.^48^

### Observations Related to Effects on Animal Health

After ingestion of the seedlings, the studied cows showed neither visible signs of illness or discomfort nor decline in milk yield throughout the observation period and thereafter, as we were informed by the owners. Nevertheless, based on the available data, subclinical changes in the organism of the animals cannot be excluded. Subsequent studies should therefore examine the defined intake of toxins, the course of concentrations of toxins in the blood, and clinical parameters indicative of a subclinical disturbance of metabolism.

The outcome is contrary to that in Père David’s deer in two Zoo’s in Germany as well as gnus in a Zoo in France which developed clinical signs with a rapid progression comparable to those also observed in horses.^5−7^ Several publications report that already relatively small amounts of maple toxins may be sufficient to poison equids.^4,14,27^ Maple toxin poisoning in horses results in muscular weakness and stiffness following respiratory depression and recumbency leading to death within 72 h^15^ Complementary myoglobinuria is also a common clinical sign in horses and was also seen in poisoned deer with fatal course.^6^

On the other hand, similar to the findings in the present study with cows, no clinical signs of poisoning were observed in studies with pastured ewes and their lambs as well as with goats exposed to sycamore seedlings. This may suggest that there might be differences in the susceptibility to toxic effects of HGA and MCPrG in some ruminant species as compared to horses.^7,32^ The susceptibility to maple toxins of species beyond horses has not yet been evaluated systematically.

After ingestion and further metabolization of HGA and MCPrG as mentioned before, MCPA-CoA causes toxicity by inhibiting acyl-CoA dehydrogenases, isovaleryl-CoA dehydrogenases, and 2-methyl-branched chain acyl-CoA dehydrogenases and thus blocking the first step of β-oxidation. MCPF-CoA inhibits enoyl-CoA hydratases in mitochondria and peroxisomes. Therefore, it has been hypothesized that both amino acids simultaneously strengthen the inhibition of β-oxidation, leading to the disruption of energy metabolism.^6^ As a result of the disturbances in β-oxidation, acyl residues that cannot be broken down further will be excreted, among others, via urine. However, MCPA and MCPF may also be further metabolized by conjugation with glycine or carnitine and excreted with urine.^9^ Therefore, the occurrence of metabolites in serum and urine has been used to confirm diagnosis of AM in horses.^4,49^

In this study, neither HGA nor MCPrG could be detected in the urine samples. Individual levels of MCPA-glycine (15,160 to 66,228 nmol/mmol of creatinine) and MCPF-glycine (561 to 1705 nmol/mmol of creatinine) detected in urine samples of all five cows on day 3 are present in Table 2. No carnitine adducts were found in the urine.

**Table 2:** Concentration of MCPrG, HGA, and Their Metabolites in Urine Samples of Study Cows (C1–C5) on Day 3

	concentration in urine (nmol/mmol of creatinine)				
item	C1	C2	C3	C4	C5
HGA^a^	−f	−	−	−	−
MCPA-G^b^	15,160	66,228	15,192	45,091	53,623
MCPA-C^c^	−	−	−	−	−
MCPrG^d^	−	−	−	−	−
MCPF-G^e^	947	561	1391	1065	1705

a Hypoglycin A (HGA) (LOD and LOQ are 166 and 546 nmol/mmol of creatinine, respectively).

b Methylenecyclopropylacetyl-glycine (MCPA-G) (LOD and LOQ are 160 and 527 nmol/mmol of creatinine, respectively).

c Methylenecyclopropylacetyl-carnitine (MCPA-C) (LOD and LOQ are 70 and 232 nmol/mmol of creatinine, respectively).

d Methylenecyclopropylglycine (MCPrG) (LOD and LOQ are 295 and 974 nmol/mmol of creatinine, respectively).

e Methylenecyclopropylformyl-glycine (MCPF-G) (LOD and LOQ are 92 and 303 nmol/mmol of creatinine, respectively).

f −, not detected.

The level of MCPA-glycine was consistently higher than that of MCPF-glycine. This corresponds with the higher concentrations of HGA compared to MCPrG in the seedlings. However, the ratio between MCPA-glycine and MCPF-glycine was not consistent between the cows and ranged between approximately 11 and 118.

The concentrations of MCPA-glycine as a metabolite of HGA in this study (Table 2) were higher than the levels that have been observed in poisoned deer (4600 und 16,800 nmol/mmol of creatinine), whereas values of MCPF-glycine (Table 2), as a metabolite of MCPrG, were lower in cows than in deer (1800 und 7500 nmol/mmol of creatinine), even though higher contents of MCPrG were detected in the seedlings of the present study (200 mg/kg) in contrast to the contents in seeds (42.9 mg/g) and leaves (0.1 mg/g) ingested by the deer.^6^ MCPA-glycine levels found in urine of poisoned horses (280–1970 nmol/mmol of creatinine) were lower than concentrations in urine of cows in the present study.^4^

Contrary to the findings in deer and horses, HGA, MCPA-carnitine, and MCPF-carnitine were not detected in urine samples of our study, which could indicate differences in absorption and/or metabolism. The fact that neither HGA nor MCPrG was detected in urine samples in this study strongly suggests that there was a rapid and complete modification in cattle contrary to the findings in deer. It has been hypothesized that the development of clinical signs of poisoning in horses and Père David’s deer may be more related to the amount of MCPrG ingested rather than with HGA due to high concentrations of MCPrG metabolites in urine samples and increasing toxic effects of AM in horses and deers.^6^ However, the toxicological relevance and role of individual maple toxins and metabolites is still not clarified.

There are different hypotheses that may explain the varying susceptibility of cattle and other species to plant toxins. Due to intense ruminal fermentation by a complex microbiome, transformation of toxins into various metabolites before absorption into the blood might occur. HGA and MCPrG represent, as amino acids, hydrophilic and soluble substances that can already be utilized by microbes in the rumen. Additionally, due to a high ruminal retention time in large ruminants, it is therefore reasonable to assume that HGA and MCPrG probably do not reach the site of their absorption, the proximal small intestine, in sufficiently high concentrations.^7,30,50^ A shorter retention time, as suspected in camelids and sheep, therefore might result in HGA absorption depending on feed availability or exposure to the toxins, as proved by the detection of HGA in serum of sheep and goats.^7,32^ In contrast, however, the toxins could have a short retention time in the rumen due to their hydrophilicity and a following quick transition to the liquid phase if they are quickly released from the plant matrix.

Still, in vitro incubation of HGA in ruminal fluid for 2 h intended to test the microbial influence on the fate of toxins showed no degradation but rather a significant increase in concentrations.^32^ Because of longer in vivo retention times in the rumen,^51^ investigations with long incubation periods are necessary to fully understand and evaluate the impact of rumen microbes on the fate of maple toxins, which has not been considered in the past. The results of this study may indicate that partial conversion of protoxins to their active forms already occurs in the rumen or post-absorptive, resulting in high concentrations on MCPA-G.

Findings of HGA in bulk tank milk samples from a farm in northern Germany led to the assumption that absorption of HGA may also occur in large ruminants^29^ depending on the extent of transformation of toxins in the foregut system. However, the detection of HGA in cows’ milk has not yet been confirmed by other studies.

Renaud et al.^7^ observed recently that some of the exposed co-grazing animals may have low concentrations of MCPA-carnitine or MCPA-glycine in serum or urine but do not show any clinical signs of poisoning. This observation of subclinical poisoning cases is in agreement with findings of Bochnia et al.^4^ Compared with diseased animals, subclinically poisoned cases had lower levels of free carnitine and acylcarnitines in serum.^7,27^

Although cows in the current study did not show any clinical signs, the influence of maple toxin intake on the energy metabolism of cows should be examined more closely, e.g., by investigating fatty acid metabolism alterations and free fatty acids in urine and serum.

By adapting to high-performance patterns regarding glucose metabolism, cattle developed a high efficiency in energy utilization in contrast to other ruminant species.^52^ It may be hypothesized that the low susceptibility of cows to the toxic effects of HGA and MCPrG may result from increased gluconeogenesis in dairy cows.^52,53^

### Milk Samples

An initial objective of the study was to identify whether there is a transfer of sycamore maple toxins into milk of dairy cows after ingestion of the seedlings. Here, we describe for the first time the occurrence of HGA and MCPrG metabolites in milk of dairy cows, demonstrating that at least metabolites may be transferred into the milk following the ingestion of HGA-/MCPrG-containing plant materials.

The levels of HGA, MCPrG, and their respective metabolites in individual milk samples are depicted in Table 3.

**Table 3:** Concentration (μg/L) of HGA and the Metabolites MCPA-G/MCPA-C and MCPF-G and Peak Areas of MCPF-C in Individual Milk Samples of Cows (C1–C5)^a^

		HGA (μg/L)^b^					MCPA-G (μg/L)^c^					MCPA-C (μg/L)^d^					MCPF-G (μg/L)^e^					MCPF-C^f^ peak area^f^				
day	time	C1	C2	C3	C4	C5	C1	C2	C3	C4	C5	C1	C2	C3	C4	C5	C1	C2	C3	C4	C5	C1	C2	C3	C4	C5
1	am	−	−	−	−	−	3.7	−	NQ	−	−	NQ	−	−	−	−	−	−	−	−	−	++	+	+	+	+
	pm	−	−	−	−	−	8.6	20	12	8.2	18	2.1	9.2	1.9	−	6.8	−	−	−	−	−	++	++	++	++	+++
2	am	−	−	−	−	−	1.4	5.2	2.1	NQ	2.5	−	NQ	−	−	NQ	NQ	NQ	−	−	−	++	++	++	++	+++
	pm	−	−	−	−	−	39	44	35	14	44	11	7.9	3.0	NQ	14	−	NQ	−	−	−	++	+++	++	++	+++
3	am	−	NQ	−	−	−	9.5	15	4.7	2.9	8.8	1.2	0.78	NQ	−	2.4	1.2	1.4	NQ	NQ	NQ	+++	+++	++	++	+++
	pm	−	−	−	−	−	34	99	50	74	51	2.9	11	2.5	4.1	7.9	NQ	−	−	−	−	+++	+++	+++	+++	++++
4	am	−	NQ	−	−	−	4.3	17	6.0	7.2	8.3	−	NQ	−	−	1.3	NQ	1.6	NQ	NQ	NQ	+++	+++	+++	+++	++++

a NQ: value below LOQ but above LOD. −, not detected.

b Hypoglycin A (HGA): LOQ = 1.12 μg/L, LOD = 0.34 μg/L.

c Methylenecyclopropylacetyl-glycine (MCPA-G): LOQ 0.99 μg/L, LOD = 0.30 μg/L.

d Methylenecyclopropylacetyl-carnitine (MCPA-C): LOQ 0.75 μg/L, LOD 0.23 μg/L.

e Methylenecyclopropylformyl-glycine (MCFP-G): LOQ 1.09 μg/L, LOD = 0.33 μg/L.

f Peak areas in milk samples: +, >1.00 × 10^4^; ++, >1.00 × 10^5^; +++, >5.00 × 10^5^; ++++, >2.00 × 10^6^.

Values of MCPrG in milk were below the limit of detection (LOD) (LOD = 2.63 μg/L). Likewise, values of HGA were either below the LOD (LOD = 0.34 μg/L) or between LOD and the limit of quantification (LOQ) (LOQ = 1.12 μg/L) (henceforward referred to as “NQ”).

Unexpectedly, traces of MCPA-glycine have already been detected in the milk of two cows (C1 and C3) before they were moved to the pasture with sycamore seedlings, indicating an exposure to maple toxins already beforehand.

Since the cows entered the pasture for the first time of the year, previous grazing on the area with sycamore seedlings can be excluded. Prior studies have noted that HGA may not be completely degraded during storage of hay and silage over a period of 8 months.^42^ However, in the present study, the grass used for feed production (e.g., silage or hay), derived from the farm’s own land, was harvested from a location far apart from the pasture with sycamore maple trees. Nevertheless, it has to be considered that the feed indeed did contain traces of HGA, e.g., from seedlings stemming from seeds that were transported over a longer distance by the wind or that a contamination of the samples occurred. Another study reported that rain water collected from seedlings may also contain measurable amounts on HGA; nevertheless, the source of the animals’ drinking water was groundwater, so water contamination is unlikely in this case.^54^

Already on day 1 of the study, milk samples from the evening milking contained quantifiable amounts of MCPA-glycine (5/5 cows) and MCPA-carnitine (4/5) in individual milk samples.

No quantifiable amounts of HGA and MCPrG as well as MCPF-glycine were found. HGB was not detected in the milk despite its presence in maple seedlings.

Therefore, we hypothesize that there may be a quick absorption and metabolism of maple toxins in dairy cows after ingestion, followed by a transfer into the milk. This could be related to a direct absorption of the non-proteinogenic amino acids and their metabolites in anterior parts of the digestive tract. There is some evidence that certain amino acids may also be absorbed prior the small intestine in the rumen.^55^ Another explanation could be a faster passage rate through the rumen to the small intestine, since the uptake of spring feed may lead to waterier, liquid chyme washing through the rumen avoiding degradation by ruminal microbes.^7^ Future studies should therefore elucidate in more detail the possibilities of gastrointestinal absorption of maple toxins.

Despite high contents of HGA in the seedlings, only the associated metabolites could be found in milk samples at low levels. On the following days of the study, MCPA-glycine was detected in individual milk samples in morning and evening milk of all 5 cows (only C4 morning milk of day 2 was NQ), while MCPA-carnitine was detected in morning (2/5, both NQ) and evening (5/5, C4 NQ) milk of day 2, morning (4/5, C3 NQ) and evening (5/5) milk of day 3, and morning milk (2/5, C2 NQ) of day 4 in individual milk samples. Values measured in evening milk where higher than in morning milk for MCPA-glycine and MCPA-carnitine. In the case of MCPA-glycine, concentrations in evening milk were on average 12.5-fold higher (36.45 μg/L) compared to morning milk (*p* < 0.004). This supports the hypothesis that there is a rapid absorption after maple toxin intake with a subsequent transfer into milk. Since cows did not graze seedlings at night, there were lower contents in morning milk compared to evening milk.

In contrast, MCPF-glycine was only detected in quantifiable amounts in morning milk of day 3 (2/5) and 4 (1/5), while there were values below LOQ in only 1/5 cows in evening milk (i.e., not detected in 4/5 cows) of day 2 and 3, suggesting differences in MCPrG metabolism or kinetics in comparison with HGA.

Additionally, what stands out in Table 3 is that there is a trend of increasing levels of MCPA-glycine over the study days. On average, MCPA-glycine concentrations in milk increased by approximately 9 μg/L per day (*p* < 0.004).

This may indicate that despite a quick metabolism of maple toxins, slight accumulation may have occurred for MCPA-glycine over the days of the experiment contrary to protoxins HGA and MCPrG. Moreover, a repeated or even increased intake with incomplete elimination of metabolites could lead to increasing levels in milk in the course of the study. Further development of clinical signs cannot be excluded in this case. Nevertheless, supplementary studies are necessary to conclusively assess the kinetics of the individual toxins, especially protoxins, including defined uptake.

Even though the direct estimation of the concentrations of MCPF-carnitine was not possible due to the lack of a reference standard, peak areas could still be used to get an impression on the increase of that metabolite over the course of the experiment. In general, the concentrations of the tentatively identified MCPF-carnitine seemed to increase in all the subsequent milk samples. In all cows, the maximum MCPF-carnitine peak areas were in samples of day 3.

The fact that HGA was measured in only two samples below LOQ in cows’ milk is contrary to the findings of Sander et al.^33^ in mare milk samples with concentrations of 0.4 μg/L HGA in a milk sample of an AM-affected horse as well as 2.4 μg/L HGA in one out of five commercial milk samples. On the other hand, elimination patterns of metabolites of the former study by Sander et al., who detected 18.5 μg/L MCPA-glycine, 24.6 μg/L MCPA-carnitine, 0.8 μg/L MCPF-glycine, and 60 μg/L MCPF-carnitine in a milk sample of an AM-affected mare as well as 1.3 μg/L MCPrG, 0.4 μg/L MCPA-carnitine, and 2.7 μg/L MCPF-glycine in one out of five commercial mares’ milk samples, available in store, are in line with our results.

It is interesting to note that values of HGA were below an LOQ of 1.1 μg/L in all milk samples contrary to the findings of Bochnia et al. 2021 (17 and 69 μg/L). In our study, HGA was detected above LOD but below LOQ in two samples from only one cow with relatively high contents on detected metabolites compared to the other cows, suggesting that concentrations of HGA in milk may be related to exposure, depending on seedling intake or diet preferences of individual cows. However, the small sample size limits such hypothesis that might be verified by long-term research in the future.

The samples from the bulk tank in our study contained only MCPA-glycine and traces of MCPA-carnitine (Table 4).

**Table 4:** Concentrations of HGA, MCPrG, and Respective Metabolites in Bulk Milk Tank Samples of Cows (Herd of 87 Cows, 5 Cows Included in the Study)^a^

day	time	HGA (μg/L)^b^	MCPA-G (μg/L)^c^	MCPA-C (μg/L)^d^	MCPrG (μg/L)^e^	MCPF-G (μg/L)^f^
1	pm	−f	5.2	NQ^g^	−	−
2	am	−	3.0	−	−	−
	pm	−	4.7	NQ	−	−
3	am	−	3.9	−	−	−
	pm	−	5.0	−	−	−
4	am	−	2.8	−	−	−

a Dotted lines show the time of emptying of the bulk tank.

b Hypoglycin A (HGA).

c Methylenecyclopropylacetyl-glycine (MCPA-G).

d Methylenecyclopropylacetyl-carnitine (MCPA-C).

e Methylenecyclopropylglycine (MCPrG).

f −, not detected.

g NQ: value below LOQ but above LOD (for MCPA-G, LOD = 0.30 μg/L; for MCPA-C, LOD = 0.23 μg/L).

Even though the rest of the herd did not have access to the pasture with maple seedlings, low levels of MCPA-glycine were detected in the bulk tank milk on all days of the experiment in the morning and in the evening with lower levels in morning than in evening milk, agreeing with the findings in individual milk samples. However, in contrast to individual milk samples from study cows, MCPA-carnitine in bulk tank milk samples was measured on days 1 and 2 in the evening above LOD but below LOQ. Of note, the bulk tank was emptied only every second days.

Still, it is important to emphasize here that the inclusion of 5 study cows was sufficient to achieve measurable concentrations of conjugated metabolites in the total tank milk that included milk of 87 lactating cows.

The conjugated metabolites should be considered as biomarkers of exposure to HGA and MCPrG. However, to date, there are no data available whether the metabolites MCPA-glycin/carnitine and MCPF-glycin/carnitine themselves have any toxicological relevance.

The observations proved that dairy cows in this study did not avoid maple seedlings during grazing. After ingestion of sycamore seedlings, metabolites appeared in milk samples of the five study cows as well as in tank milk of the whole herd in less than 12 h. Metabolites were also detectable in the urine of cows. These findings were accompanied by the absence of any clinical signs or discomfort in the animals. Therefore, it seems that cows quickly metabolized and excreted HGA and MCPrG and have a low or completely missing susceptibility to HGA poisoning as it is also known for small ruminants compared with the highly susceptible horses. Moreover the study provides a basis for following investigations on suspected ruminal transformations. Despite its exploratory nature, this study offers some insights into the transfer behavior of maple toxins into the milk. It was shown that under a typical setting for maple uptake on a pasture, the transfer of the toxins HGA and MCPrG themselves into milk may be negligible. However, conjugated metabolites are transferred to the milk in less than 12 h. To quantitatively evaluate this transfer of maple toxin metabolites into milk, further data are required.

## Abbreviations

AM: atypical pasture myopathy;
BfR: German Federal Institute for Risk Assessment
CoA: coenzyme A
DM: dry matter
FW: fresh weight
HGA: hypoglycin A
HGB: hypoglycin B
MCPA: methylenecyclopropylacetyl
MCPrG: methylenecyclopropylglycine
NRL: National Reference Laboratory
LC/MS–MS: liquid chromatography–tandem mass spectrometry
LC–HR-MS: liquid chromatography–high-resolution mass spectrometry
VDLUFA: Association of German Agricultural Analytic and Research Institutes
